# Novel Dolutegravir and Lenacapavir Resistance Patterns in Human Immunodeficiency Virus Type 2 Infection: A Case Report

**DOI:** 10.1093/ofid/ofae705

**Published:** 2024-11-29

**Authors:** Jeroen J A van Kampen, Els van Nood, Rizwan Mahmud, Zoë Krullaars, Tess Voskamp, Mike Voskamp, Tess Nijssen, Jolanda J C Voermans, Charlotte Charpentier, Quentin Le Hingrat, David A M C van de Vijver, Rob A Gruters, Thibault Mesplède

**Affiliations:** Viroscience Department, Erasmus MC, Rotterdam, The Netherlands; Department of Medical Microbiology & Infectious Diseases and Department of Internal Medicine, Erasmus MC, Rotterdam, The Netherlands; Viroscience Department, Erasmus MC, Rotterdam, The Netherlands; Viroscience Department, Erasmus MC, Rotterdam, The Netherlands; Viroscience Department, Erasmus MC, Rotterdam, The Netherlands; Viroscience Department, Erasmus MC, Rotterdam, The Netherlands; Viroscience Department, Erasmus MC, Rotterdam, The Netherlands; Viroscience Department, Erasmus MC, Rotterdam, The Netherlands; Service de Virologie, INSERM, IAME, UMR 1137, AP-HP, Hôpital Bichat-Claude Bernard, Université Paris Cité, Paris, France; Service de Virologie, INSERM, IAME, UMR 1137, AP-HP, Hôpital Bichat-Claude Bernard, Université Paris Cité, Paris, France; Viroscience Department, Erasmus MC, Rotterdam, The Netherlands; Viroscience Department, Erasmus MC, Rotterdam, The Netherlands; Viroscience Department, Erasmus MC, Rotterdam, The Netherlands

**Keywords:** bictegravir, dolutegravir, drug resistance mutation, HIV-2, lenacapavir

## Abstract

**Background:**

The treatment management of human immunodeficiency virus (HIV)-2 infection presents greater challenges compared to HIV-1 infection, primarily because of inherent resistance against non-nucleoside reverse transcriptase inhibitors. Integrase strand transfer inhibitors, particularly dolutegravir, have improved treatment outcomes for people with HIV-2. Lenacapavir, a novel and potent antiretroviral capsid inhibitor, offers additional therapeutic options. However, limited knowledge exists regarding HIV-2 resistance against dolutegravir and lenacapavir.

**Methods:**

We report the case of a treatment-experienced individual who did not achieve virological suppression with regimens containing dolutegravir and lenacapavir. Clinical monitoring, genotypic and phenotypic resistance assays, and *in silico* structural modeling were performed.

**Results:**

Lenacapavir was added to a failing regimen of boosted darunavir, twice daily dolutegravir, and 2 nucleoside reverse transcriptase inhibitors. Initially, this addition led to a decline in the viral load and increase in CD4+ T-cell count, despite the identification of a previously unreported combination of integrase resistance mutations. However, virological suppression was not achieved and viral load, although reduced, resumed increasing. This rebound was associated with the development of an N73D capsid substitution in HIV-2, which conferred resistance against lenacapavir. Based on cell-based assays predicting hypersusceptibility to bictegravir, the regimen was adjusted to oral lenacapavir plus bictegravir/emtricitabine/tenofovir alafenamide, resulting in a resumption in viral load decline.

**Conclusions:**

Although lenacapavir demonstrated therapeutic potential, our case underscores the critical need to combine it with other fully active antiretroviral agents to prevent the rapid emergence of resistance and achieve long-term virological control in treatment-experienced individuals with HIV-2.

## Teaser

HIV-2 treatment options are limited, and resistance mechanisms to integrase and capsid inhibitors are understudied. Despite emerging resistance, lenacapavir shows promise in salvage therapy, optimally when combined with drugs that have high barriers to resistance like dolutegravir or bictegravir.

## Graphical Abstract

**Graphical abstract:**
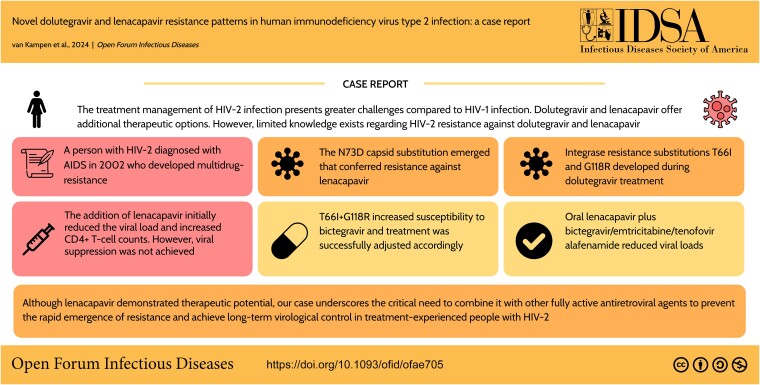
This graphical abstract is also available at Tidbit: https://tidbitapp.io/tidbits/novel-dolutegravir-and-lenacapavir-resistance-patterns-in-human-immunodeficiency-virus-type-2-infection-a-case-report?utm_campaign=tidbitlinkshare&utm_source=IO

## BACKGROUND

An estimated 1 to 2 million people live with human immunodeficiency virus type 2 (HIV-2) worldwide, mostly in Western Africa [1]. Despite a slower disease progression than HIV-1, untreated HIV-2 eventually leads to AIDS in the majority of people [2]. Managing HIV-2 presents unique challenges compared to HIV-1. One obstacle is the scarcity of diagnostics tools to discriminate between HIV-1, HIV-2, and HIV-1/HIV-2 double infections [3]. Additionally, the treatment of HIV-2 is complicated by the virus natural resistance to some antiretroviral drugs, including the entire class of nonnucleoside reverse transcriptase inhibitors and some protease inhibitors [4, 5]. Even the drugs that are effective against HIV-2 show reduced potency when compared to HIV-1 [6–8]. Furthermore, treatment optimization based on HIV-2 genotypic resistance analyses is impeded by insufficient knowledge of the specific mutations that drive antiretroviral resistance. Recently, the discovery of second-generation integrase inhibitors, such as dolutegravir and bictegravir, has offered new opportunities to facilitate the optimization of HIV-2 treatment [9]. Knowledge about HIV-2 clinical resistance against these two drugs remains incomplete despite recent studies examining the *in vitro* drug susceptibility of many HIV-2 mutations and combinations of mutations [10, 11].

Lenacapavir is a first-in-class capsid inhibitor active against HIV-1 and HIV-2, although it is less active against the latter [12]. Its inception also offered avenues to improve HIV-2 treatment. However, there is little information regarding HIV-2 resistance mutations against this new drug [13]. We report here novel resistance mutations in a person with HIV-2 experiencing treatment failure with regimens that included dolutegravir (DTG) and lenacapavir. These mutations were validated as *bona fide* resistance mutations through site-directed mutant analyses, and resistance was interpreted using *in silico* structural modeling.

## MATERIALS AND METHODS

### Case Report

We describe the case of an HIV-2–positive person who was diagnosed in 2002 with AIDS and developed a multidrug-resistant HIV-2 virus over several years due to repeated virological failure and therapy changes. Routine laboratory tests included CD4+ T-lymphocyte counts and HIV-2 RNA loads measured using quantitative polymerase chain reaction with a 50 RNA copies/mL of blood lower limit of quantification.

### Patient Consent Statement

Institutional ethics approval was obtained for data collection (METC 2015-566), and the person provided written informed consent for the anonymous publication of their clinical data.

### Infectivity and Drug Susceptibility Assays

The pROD10 HIV-2 group A proviral plasmid (GenBank ID: KY272752) was used for site-directed mutagenesis to produce the pROD10_T66I_, pROD10_G118R_, pROD10_T66I + G118R_, and pROD10_N73D_ plasmids using a previously described protocol [14]. Wild-type (WT) and mutated plasmids were transfected into 293T cells to produce the WT, T66I, G118R, T66I + G118R, and N73D HIV-2 viruses [14]. Viral stocks were quantified by RNA copy number, measured via quantitative polymerase chain reaction against a standard curve. The relative infectiousness of WT, T66I, G118R, T66I + G118R, and N73D was tested using the TZM-bl reporter cells and expressed as half-effective concentrations (EC_50_s)—the viral concentrations required to induce half of the maximal reporter signal [14]. A higher EC_50_ value indicates reduced infectivity, as more viral particles (measured by viral RNA) are needed to reach half of the maximal reporter signal. We also reported the maximal reporter signal recorded for each virus relative to the WT virus, expressed as a percentage and termed maximal infectivity. A decrease in maximal infectivity compared to WT indicates that the virus induces lower luciferase production from the TZM-bl reporter cells even at saturating numbers of virions. For resistance assays, we used serial dilutions of dolutegravir (2.5 μM to 1 pM), bictegravir (1.9 μM to 0.9 pM), raltegravir (2.5 μM to 1 pM), elvitegravir (EVG) (2.5 μM to 1 pM), cabotegravir (750 nM to 0.4 pM), and lenacapavir (714 nM to 1 fM) from MedChemExpress [14]. Relative fluorescence units were measured on an Infinite 200 PRO plate reader (Tecan), normalized, and plotted against log-transformed RNA viral copies for the infectivity assay or drug concentrations for the drug susceptibility assay with GraphPad Prism v 9.4.1 (GraphPad Software, LLC). The half-maximal inhibitory concentrations (termed IC_50_) of each mutant HIV-2 were calculated and are reported with their 95% confidence intervals. In addition, fold changes relative to WT are also reported. All data points were produced in triplicates and experiments were repeated at least twice with 2 different viral batches. As we performed phenotyping while the case developed, assays with integrase and capsid mutations were conducted separately, always using a contemporaneously produced WT virus as a control and for normalization.

### In Silico Structural Modeling

The WT and mutated HIV-2 integrase proteins were modeled with I-TASSER as published [15–17], using a structure of the HIV-1 intasome in complex with the integrase inhibitor bictegravir and DNA, PDB ID:6PUW [18], as a guide. We chose this template because it contains specific changes that occur only when the protein is bound to the DNA and inhibitor. These changes are not easily modeled in a *de novo* structure. The top-ranking I-TASSER structures were further refined with ModRefiner [19]. HIV-2 and HIV-1 integrase structures were compared using the Pairwise Align tool of the RCS PDB. *In silico* drug binding was performed with AceDock [20], as published previously [14]. The binding of EVG and raltegravir to integrase was further informed by examining the structure of the prototype foamy virus integrase binding to these drugs, PDB ID:3L2U and 3OYA, respectively [21]. Molecular interactions between proteins and ligands were evaluated with Maestro v.13.2.128 (Schrodinger LLC). PyMol was used for visualization and image production (Schrodinger LLC).

## RESULTS

**Figure 1.**
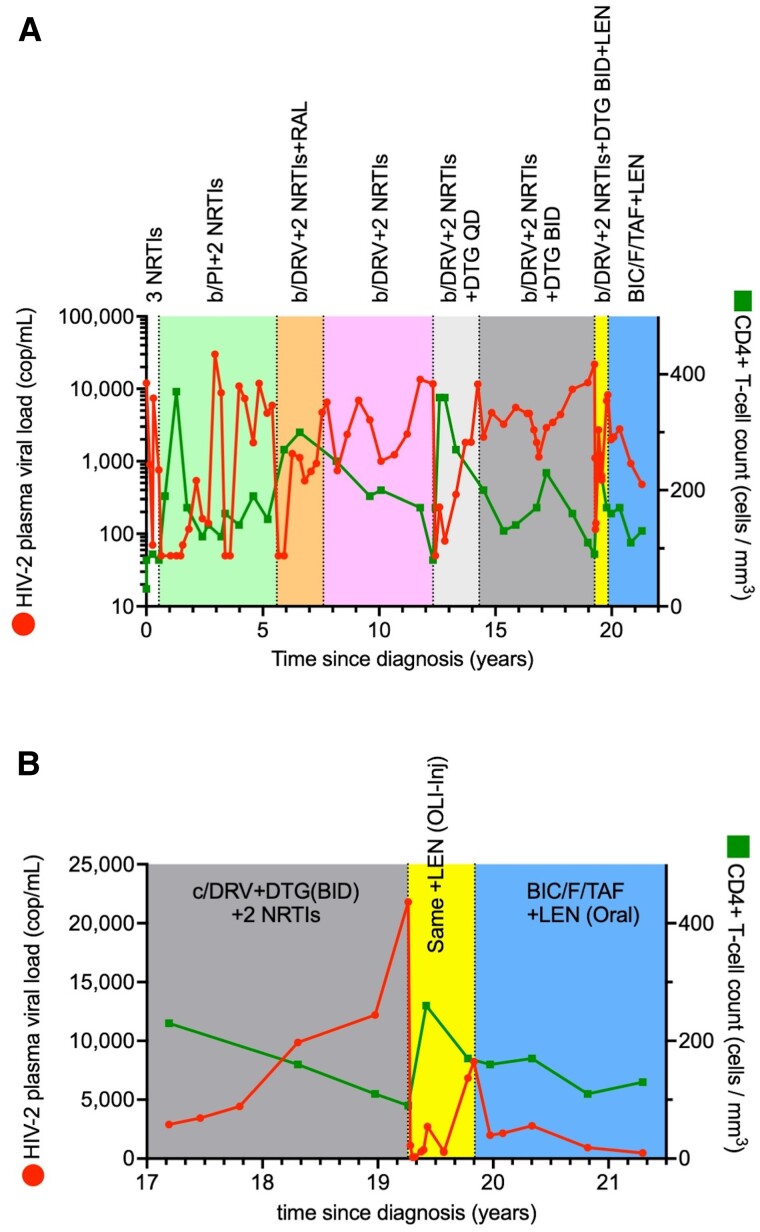
CD4+ T-cell counts and viral loads during the treatment course. The plasma HIV-2 viral loads are shown in red dots. CD4+ T-cell counts are shown in green squares. (*A*) Complete overview of the treatment course. The following cART regimens are shown at the top of the graph: 3 NRTIs (white), boosted PI + 2 NRTIs (green), boosted-DRV + RAL + 2 NRTIs (orange), boosted-DRV + 2 NRTIs (pink), boosted-DRV + DTG (50 mg) + 2 NRTIs (light gray), boosted DRV + DTG (100 mg) + 2 NRTIs (gray), boosted-DRV + DTG (100 mg) + 2 NRTIs + lenacapavir oral lead-in followed by subcutaneous administration (yellow), and BIC + 2 NRTIs + lenacapavir oral administration (blue). (*B*) Recent treatment course. The following cART regimens are shown at the top of the graph: DRV/c + DTG (100 mg) + 2 NRTIs (grey). DRV/c + DTG (100 mg) + 2 NRTIs + lenacapavir oral lead-in followed by subcutaneous administration (yellow), and BIC + 2 NRTIs + lenacapavir oral administration (blue).

An adult female was admitted to our hospital because of a *Pneumocystis jirovecii* pneumonia and cytomegalovirus retinitis in 2002. She tested positive for HIV-2 (immunoblot positive for HIV-2 and HIV-2 plasma viral load of 1·2 × 10^4^ RNA copies/mL) and negative for HIV-1 (immunoblot negative for HIV-1 and undetectable HIV-1 plasma viral load). Her CD4+ T-cell count was 30 cells per microliter. The complete treatment course is illustrated in Figure 1. Combined antiretroviral therapy (cART) was commenced, first with a combination of 3 nucleot(s)idic reverse transcriptase inhibitors (NRTIs: lamivudine, tenofovir disoproxil fumarate, and didanosine) and then with a ritonavir-boosted protease inhibitor with 2 NRTIs when viral suppression was not achieved. The reason for initiating therapy with 3 NRTIs was not recorded in the patient dossier. However, we speculate that, at the time, only limited evidence existed concerning the activity of lopinavir/ritonavir against HIV-2, since this combination was approved for HIV-1 treatment only after 2000. Viral load suppression was achieved temporarily with the boosted protease inhibitor regimen, but virological failure ensued. After 5.6 years of therapy, the regimen was changed to ritonavir-boosted darunavir, raltegravir, abacavir, and lamivudine to include darunavir that had shown greater potency and raltegravir that had just received approval for the treatment of HIV-1 and demonstrated effectiveness against HIV-2 *in vitro*. Viral suppression was achieved once again transiently before treatment failure. However, raltegravir was discontinued 2 years later (2010), as it had no significant impact on HIV-2 plasma viral load nor CD4+ T-cell counts, which continued to decline (years 7.6–12.3) (Figure 1*A*).

The second-generation integrase strand-transfer inhibitor (INSTI) dolutegravir (initially 50 mg daily, subsequently increased to 50 mg twice daily) was added to the same regimen in 2015 and the booster for darunavir was switched from ritonavir to cobicistat. However, the addition of DTG failed to sustainably reduce the HIV-2 plasma viral load or improve CD4+ T-cell counts: the HIV-2 plasma viral load rose to 2.2 × 10^4^ copies/mL, the CD4 T-cell count dropped to 90 cells/µL, and the T66I and G118R integrase substitutions emerged (Table 1). Consequently, the cART regimen was modified to include lenacapavir on a compassionate use basis (Figure 1*B*). Oral lead-in was tolerated without side effects. Subsequently, subcutaneous lenacapavir was administered as recommended. In the months following the first injection, the patient experienced pain in the left leg, and a diagnosis of bursitis was made, deemed unrelated to the administration of lenacapavir. Initially, the lenacapavir-containing regimen reduced HIV-2 plasma viral loads (115 copies/mL) and increased CD4 counts (to 260 cells/µL). However, this improvement was not maintained. Forty-one days after lenacapavir initiation, the N73D substitution in the capsid emerged. Notably, this substitution was not detected in several samples collected before lenacapavir exposure nor from cerebrospinal fluid taken 5 days apart from a blood sample that yielded the same mutation. Based on our phenotypic susceptibility results with the T66I + G118R mutant (Table 2), the patient was switched to bictegravir instead of dolutegravir. Darunavir was removed and emtricitabine/tenofovir alafenamide and lenacapavir were continued, the later was switched to weekly oral administration both to achieve higher plasma levels and because the patient was not willing to receive another injection. Throughout the >21 years of treatment, this was the only adjustment made due to tolerance or toxicity; all other treatment changes were implemented due to viral load detectability. Lenacapavir use was maintained despite the emergence of N73D because the same mutation in HIV-1 (N74D) has been shown not to limit clinical benefits of lenacapavir during functional monotherapy. This switch to bictegravir/F/TAF and oral lenacapavir was associated with a modest viral load decrease from 8.23 × 10^3^ to 1.99 × 10^3^, marking a reversal of the rising trend in HIV-2 plasma viral loads (6.3 × 10^2^; 6.84 × 10^3^; 8.23 × 10^3^). The 2 most recent viral loads were 9.27 × 10^2^ and 4.78 × 10^2^, confirming the overall positive effect of this regimen change on viral suppression. However, CD4+ T-cell counts were only maintained and failed to improve significantly (Figure 1*B*).

**Table 1.** List of Resistance Mutations 2003–2020

Sampling Year^a^	Reverse Transcriptase	Protease	Integrase^b^
2003	**M184 V,** K223R	None detected	ND
2008	**Y115F, M184 V,** K223R	**I50 V, I54L, I82F,** V10I, T56 V, V71I	None detected
2014	**Y115F, M184 V,** F214FL, K223R	**I50 V, I54L, I82F,** V10I, T56 V, I64 V, V71I	None detected
2016	**Y115F, M184 V,** K223R	**I50 V, I54L, I82F,** V10I, T56 V, I64 V, V71I	**T66I, G118R**
2018	**Y115F, M184 V,** K223R	**I50 V, I54L, I82F,** V10I, T56 V, I64 V, V71I	**T66I, G118R**
2019	**Y115F, M184 V,** K223R	**I50 V, I54L, I82F,** V10I, T56 V, I64 V, V71I	**T66I, G118R**
2020	**K70Q, Y115F, M184 V,** V111I, K223R, D67N	**I50 V, I54L, I82F,** V10I, T56 V, I64 V, V71I	**T66I, G118R**

Sequences were analyzed with the Stanford HIVdb Program for HIV-2 (last accessed on 17 April 2024) for major and accessory resistance mutations. Major resistance mutations are shown in bold. Accessory resistance mutations are shown as plain text. Subsequently, sequences were analyzed with the HIV-2 genotypic drug resistance interpretation algorithm of the ANRS (accessed on 17 April 2024). Additional mutations scored by this algorithm (mutations associated with resistance and mutations associated with possible resistance) were added and are shown as underlined text.

Abbreviation: ND, not determined.

^a^Natural capsid polymorphisms: H4Q, G6A, I12 V, E78D, V81A, Q82A, E96D/E, I113I/V P119A, I152 V, P159S, G224T.

^b^Natural integrase polymorphisms: K4R, V19I, S23A, N30 K, S39T, A41P, K46 K/R, L72I, E92E/G, I133 V, S138T, A153S, I180 V, I259 V.

**Table 2.** Infectivity and Resistance of WT, T66I, G118R, and T66I + G118R HIV-2

	Infectivity		Resistance									
Genotype	Relative EC_50_ [95%CI]	Relative maximal infectivity [95% CI]	DTG		BIC		RAL		EVG		CAB	
			IC_50_ in nM [95% CI]	FC	IC_50_ in nM [95% CI]	FC	IC_50_ in nM [95% CI]	FC	IC_50_ in nM [95% CI]	FC	IC_50_ in nM [95% CI]	FC
WT	1 [0.9–1.1]	100% [97–104%]	0.4 [0.3–0.6]	1	0.3 [0.2–0.3]	1	1 [0.1–5]	1	1.5 [0.1–4]	1	0.2 [0.2–0.3]	1
T66I	1.3 [1.2–1.3]^a^	76% [73–79%]^a^	0.1 [0.1–0.2]^a^	0.4	0.1 [0.1–0.1]^a^	0.4	414 [233–764]^a^	>200^a^	0.1 [0.01–0.3]	0.07	0.02 [0.02–0.02]^a^	0.1
G118R	1.7 [1.2–3.5]^a^	43% [36–61%]^a^	11.2 [5.4–31]^a^	29	1.9 [1.5–2.2]^a^	7	1582 [809-max]^a^	>200^a^	2 [1–3.4]	1.4	2.5 [1.1–5.2]^a^	12
T66I/G118R	2 [1.2–10.3]^a^	10% [8–22%]^a^	292 [43-max]^a^	>200	0.05 [0.04–0.07]^a^	0.2	1180 [291-max]^a^	>200^a^	124 [43–489]^a^	85.2	0.1 [0.01–0.2]	0.7

Abbreviations: EC_50_, half-effective concentrations of virus; FC, fold-change in drug susceptibility; IC_50_, half-inhibitory concentration of drug.

^a^ *P* < .05, t-test.

The impact of the T66I and G118R substitutions on HIV-2 susceptibility to INSTIs was initially unclear. At the time, several drug susceptibility prediction algorithms did not score these mutations as potentially conferring HIV-2 resistance against INSTIs (HIV grade: http://www.hiv-grade.de/HIV2EU; and ANRS-MIE interpretation database: https://hivfrenchresistance.org/hiv-french-resitance-hiv-2/) [22]. Despite the previous report of an isolated G118R substitution in HIV-2 emerging under raltegravir, G118R had not been shown to confer resistance against INSTIs in HIV-2 at that time [23]. To date (6 November 2024), HIV grade scores T66I or G118R in HIV-2 as susceptible to raltegravir, EVG, and DTG. The ANRS-MIE platform reports G118R as a resistance mutation associated with resistance against EVG, DTG, CAB, and bictegravir, while T66I is not listed as a potential resistance mutation. To address this knowledge gap, we synthesized site-directed mutants of HIV-2 featuring T66I, G118R, and the combined T66I + G118R mutations and conducted infectivity and INSTI phenotypic susceptibility tests (Table 2). First, these mutations were found to decrease viral infectiousness. The reduction in viral infectivity was more pronounced than what could be inferred from the changes in relative EC_50_ values alone (all below a 2-fold change). Specifically, the T66I and T66I + G118R mutations reduced maximal infectivity to 78% and 10% of the wild type (WT), respectively. The G118R mutation alone showed an intermediate effect, with maximal infectivity at 44% of WT. Second, resistance assays revealed that the T66I mutation exhibited high resistance (fold change > 200) against raltegravir but, surprisingly, increased the *in vitro* susceptibility to the other integrase strand transfer inhibitors. In contrast, the G118R mutation alone conferred resistance to all INSTIs but EVG. Intriguingly, when combined with T66I, this resistance profile shifted, showing hyper-susceptibility to bictegravir and cabotegravir but maintaining high resistance (raltegravir) or increasing resistance levels (dolutegravir and EVG) against other INSTIs. This finding aligns with the clinical emergence of T66I + G118R during dolutegravir exposure (Table 1).

**Figure 2.**
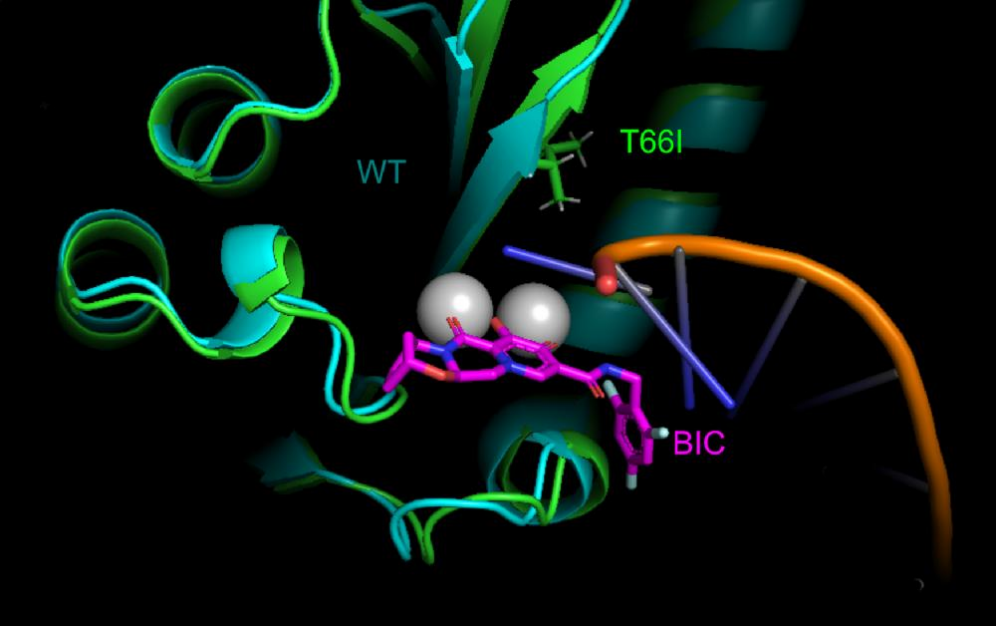
Structural modeling of differences between HIV-2 WT and T66I integrase proteins. The WT protein is shown in teal, and the T66I mutant is in green. Mg^2+^ ions appear in gray. The viral DNA and bictegravir (BIC) are illustrated.

Intrigued by these observations, we analyzed the *in-silico* structure of HIV-2 integrase to understand the divergent effects of T66I on different drugs. It has been noted before that, in HIV-1, T66 interacts with one of the two Mg^2+^ ions that serve in the strand transfer reaction [24]. The T66I substitution in HIV-1 may apply a steric hindrance to the second Mg^2+^ ion and potentially remodel the catalytic site [24]. With this information in mind, we made two observations that help to understand why T66I confers resistance against EVG in HIV-1 but not HIV-2. First, T66I in HIV-1 indeed points towards the second Mg^2+^ ion within the catalytic site. The likely movement imposed by the substitution to this ion may be particularly detrimental to EVG versus DTG, bictegravir, or CAB because EVG coordinates the two Mg^2+^ ions via its single 3-carboxylic acid. The possible involvement of a second oxygen linked to the same pyridine ring was not apparent in our docking results. In contrast, DTG, bictegravir, and CAB coordinate the two ions via two oxygens and one hydroxyl group distributed over two pyridine rings. This increased reach likely makes them less sensitive than EVG to the remodeling of the catalytic core imparted by T66I in HIV-1. Second, T66I in HIV-2 does not point towards the second Mg^2+^ ion but rather elongates, rigidifies, and reorients the 1st beta-strand, on which D64 is located (Figure 2). This change may pull the divalent ions further inside the catalytic pocket and negatively impact raltegravir binding because of the pi-stacking of the oxadiazole ring of raltegravir with Y143 [25], which impairs its ability to adjust further within the catalytic site. In contrast, the other INSTIs, including the smaller EVG, may accommodate this change well.

The impact of the N73D substitution on the susceptibility of HIV-2 to lenacapavir was also unknown at the time. To investigate this, we engineered a site-directed mutant of HIV-2 containing the N73D substitution in the capsid. The production of N73D HIV-2 stock was consistently 2.5-fold lower than the WT virus as measured by the amount of RNA copies recovered from 293T cell culture fluids post-transfection (data not shown). Additionally, the maximal infectivity of the HIV-2 carrying the N73D substitution was lower than that of the WT (66% vs 100%, respectively, Table 3). N73D conferred high resistance levels against lenacapavir (Table 3).

**Table 3.** Infectivity and Resistance of WT and N73D HIV-2

	Infectivity		Resistance	
Genotype	Relative EC_50_ [95% CI]	Relative maximal infectivity [95% CI]	IC_50_ in nM [95% CI]	FC
WT	1 [0.97–1]	100% [99–102%]	0.3 [0.3–0.3]	1
N73D	0.9 [0.6–1.5]	66% [55–91%]^a^	16.5 [15–18]^a^	55

Abbreviations: EC_50_, half-effective concentrations of virus; FC, fold-change in drug susceptibility; IC_50_, half-inhibitory concentration of drug (lenacapavir); WT, whild-type.

^a^ *P* < .05, t-test.

## DISCUSSION

Our case highlights the complexities of managing HIV-2 infection, especially in patients who have experienced the development of resistance mutations against newer drugs. The limited treatment options can be quickly exhausted, stressing the need to carefully avoid the development of adherence-related resistance. In the case reported here, the introduction of lenacapavir appeared to stabilize the infection, contributing to the subsequent partial success with the bictegravir/emtricitabine/tenofovir alafenamide regimen. We speculate that mutations emerging during treatment failure with DTG potentially aided this partial treatment success by diminishing the retroviral fitness.

Comparative molecular virology reveals both similarities and differences between HIV-1 and HIV-2 in terms of infectivity and resistance. Specifically, the T66I substitution in HIV-2 integrase reduced infectivity to levels similar to those observed with T66I in HIV-1 (1.3 vs 1.13-fold, respectively) [26]. However, G118R had a more significant impact on infectivity in HIV-1 (2.8-fold [14],) than HIV-2 (1.7-fold, Table 2). Reciprocally, maximum infectivity was unchanged by the G118R substitution in HIV-1 but decreased in HIV-2. How these changes in infectivity may translate into fitness differences in primary cells was not tested. Because of donor variability, peripheral blood mononuclear cell-based assays are difficult to standardize [27], thus immortalized reporter TZM-bl cells are used in most infectivity and resistance assays [10, 11, 14, 26, 28].

Previous studies have shown that the T66I substitution can increase susceptibility to some integrase strand transfer inhibitors in HIV-1 [28]. We confirmed the increased susceptibility of T66I-HIV-1 to dolutegravir inhibition and further showed that the T66I + R263K combination of substitutions was also hypersusceptible to dolutegravir [26]. Here, we observed similar results with HIV-2, with T66I conferring increased susceptibility to dolutegravir and cabotegravir (Table 2). In addition, T66I also increased HIV-2 susceptibility to bictegravir.

Interestingly, while T66I conferred resistance to raltegravir in both HIV-1 and HIV-2 [26], the resistance levels varied significantly between them, with low (2.4-fold) in HIV-1 versus high (>200-fold) in HIV-2. In addition, the resistance phenotype to EVG was inverted compared to HIV-1, whereby T66I in HIV-1 was resistant to EVG [26, 28], but T66I in HIV-2 was hypersusceptible to EVG inhibition.

Similar to HIV-1, the G118R substitution in HIV-2 integrase resulted in resistance to all INSTIs [29], although this effect was modest and did not reach significance for EVG (Table 2). The combination of T66I and G118R in HIV-2 rendered the virus susceptible to bictegravir, an aspect yet to be explored in HIV-1. This led us to switch the person's treatment to a bictegravir-based regimen, resulting in reduced HIV-2 plasma viral loads. This partial response aligns with our cell-based resistance findings, although the molecular mechanisms underlying the resistance against DTG versus susceptibility to BIC remain enigmatic. Our structural in silico modeling helped to explain the other results of our resistance assays.

Our results concerning the N73D substitution in the capsid of HIV-2 conferring resistance against lenacapavir were clearer. The same substitution (N74D) was found to emerge during cell-based selection experiments and also to confer 22-fold resistance against lenacapavir in HIV-1 while also reducing fitness [30, 31]. It is important to note that our assays are designed to measure infectivity and not fitness. Specifically, luciferase production by TZM-bl cells provides an estimate of postentry efficiency. The N74D substitution in HIV-1 has been shown to affect nuclear entry [32] and reduce single-cycle infectivity by 50% in an assay comparable to ours [30]. In line with this, our results showed a 44% reduction in maximal infectivity. We also note that capsid inhibition in virus-producing cells was associated with the formation of viral particles with abnormal shapes [33]. However, our assays were not designed to measure the impact of N73D on viral production inhibition by lenacapavir. We, however, reported that the production of N73D by transfected 293T cells was consistently lower than that of the WT virus in the absence of lenacapavir. Given the sequential nature of infection and viral production, a multicycle infectivity assay might provide a clearer picture of the overall levels of resistance against lenacapavir imparted by N73D and its impact on fitness. In comparable assays, we observed higher resistance levels with N73D in HIV-2 than results with HIV-1 reported by others [30]. Some of this disparity might be attributable to difference in assays design, as previously reported [13].

In conclusion, our study reports a novel resistance mutational pathway in HIV-2 integrase and documents resistance development against lenacapavir in this virus. Resistance mutations at failure with integrase inhibitors can emerge from HIV-1 and HIV-2. The differences in resistance patterns between HIV-1 and HIV-2 observed here highlight that resistance data cannot always be directly extrapolated from HIV-1 to HIV-2. Thus, HIV-2 resistance against integrase inhibitors, particularly dolutegravir is worth studying further. Given the current dolutegravir rollout in countries in which HIV-2 is circulating, we believe it essential that persons with HIV-2 treated with DTG-based regimens be closely monitored for treatment failure. Virological failure may have more severe consequences for people with HIV-2 than HIV-1 because of the more rapid selection of resistance and limited treatment options. Reciprocally, lenacapavir may provide additional treatment opportunities for people with HIV-2 in Western countries, but its current narrow clinical approval profile and high cost may limit its applicability in developing countries.

## Notes

***Acknowledgments.*** We want to thank Gilead Sciences for providing lenacapavir on a compassionate use basis. The following reagents were obtained through the NIH HIV Reagent Program, Division of AIDS, NIAID, NIH: Human Immunodeﬁciency Virus 1 (HIV-1), Strain NL4-3 Infectious Molecular Clone (pNL4-3), ARP-2852, contributed by Dr. M. Martin; and TZM-bl Cells, ARP-8129, contributed by Dr. John C. Kappes and Dr. Xiaoyun Wu. The HIV-2 infectious molecular clone pROD10 was obtained from the National Institute for Biological Standards and Control (#0232), contributed by Dr. J-M Bechet [34].

***Financial support*.** This work was supported by the Dutch Aidsfonds [grant P-60804] to R.G., the Canadian Institutes for Health Research [HB1 164063] to T.M., and the National Institute of Allergy and Infectious Diseases [grant number R01AI147330] to D.v.d.V. The funding sources played no role in the collection and analysis of the data, writing of the report, or decision to publish.
